# Multidisciplinary Approach to Treatment of Midline Diastema With Edge-to-Edge Bite

**DOI:** 10.7759/cureus.30400

**Published:** 2022-10-17

**Authors:** Sumukh Nerurkar, Ranjit Kamble, Japneet Kaiser, Jeni Mathew

**Affiliations:** 1 Department of Orthodontics, Sharad Pawar Dental College, Datta Meghe Institute of Medical Sciences, Wardha, IND

**Keywords:** midline diastema, spacing, frenectomy, oral medicine and periodontology, orthodontics

## Abstract

Aesthetic treatments have gained massive popularity in the recent past. Patients with midline diastema and spacing are among the most common complaints reported to an orthodontic clinic. The major complaint with such cases is the poor aesthetics that accompany them. Although many restorative treatment options are available to treat these cases, their long-term success is still questionable. The primary aetiology is abnormal frenal attachment, as seen in the case. Getting rid of the etiologic factor is vital to attain a stable treatment. In the present case, a frenectomy was performed to correct the abnormal frenal attachment. Even after correcting the aetiology, correct retention protocol is equally essential. The present article presents the treatment of a case with midline diastema and an edge-to-edge bite, and a high frenal attachment.

## Introduction

Midline diastema or spacing is one of the most common complaints of patients or parents and is one of the main reasons for undergoing orthodontic treatment. Although it is a normal finding in children of around 11-13 years and is known as the ugly duckling stage. It is considered abnormal only when it persists after this age [1]. A spacing is known as a midline diastema if the spacing between the mesial surface of central incisor teeth is more than 0.5mm. A prevalence of 14.8% was found in the maxillary arch. According to Angle, the most common cause of midline diastema is abnormal frenal attachment. The presence of mesodens and arch size-tooth size discrepancy, missing lateral incisors are various other reasons for spacing [2,3]. According to Kaimenyi, 50% of the midline diastema in the maxillary arch was associated with papillary penetrating frenal attachment [4]. In cases where diastema is related to abnormal frenum, frenectomy has to be performed to avoid relapse. There are two schools of thought about the timing of frenectomy. Some clinicians believe it must be done at the start of the treatment, while others believe it must be performed after the conclusion of orthodontic tooth movement. The latter believe that performing a frenectomy before the commencement of treatment may cause fibrosis and reduce the rate of tooth movement [5]. The present case describes the multidisciplinary treatment of a patient with midline diastema with papilla penetrating frenal attachment with an edge-to-edge bite [6,7]. 

## Case presentation

**Figure 1:**
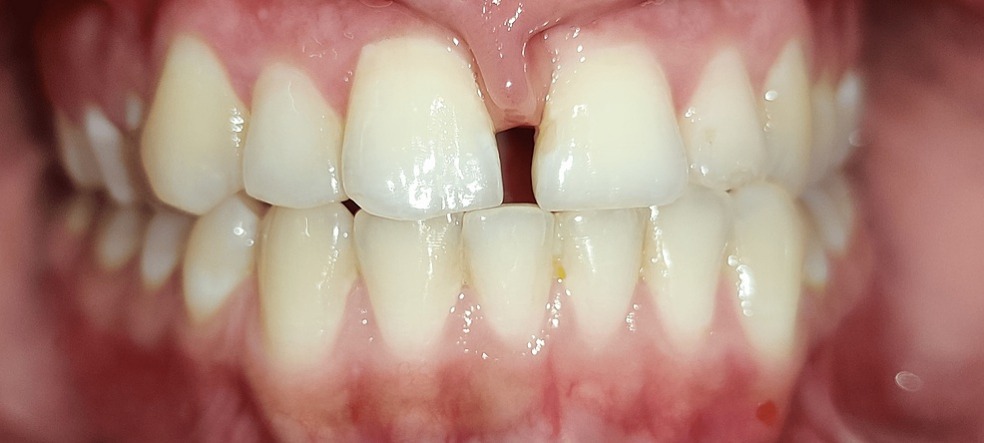
Midline Diastema and Frenum

**Figure 2:**
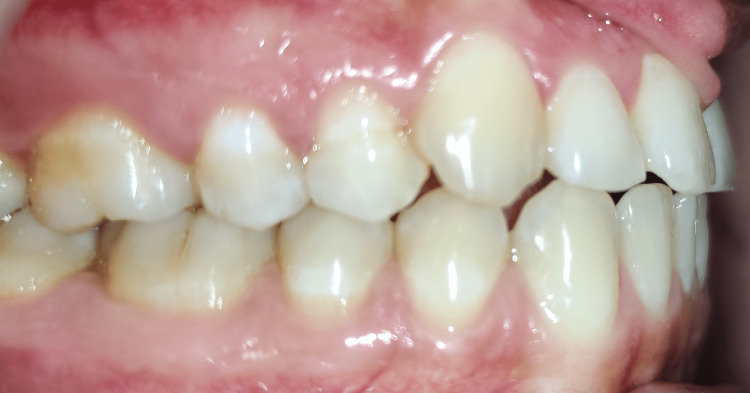
Molar relation and edge-to-edge bite

A 22-year-old female reported to the orthodontic OPD with the chief complaint of a gap in the upper front teeth. The patient had a bilaterally symmetrical face, competent lips, average depth of mento-labial sulcus, average nasolabial angle and a straight profile. The patient revealed no past medical or dental history. The orthopantomogram (OPG) was used to rule out the presence of any unerupted mesodens. Intra-oral examination revealed a diastema of 4mm. A papilla-penetrating frenal attachment. The molar relation was a super class I on both sides. The overjet and overbite were reduced. The patient had a thick lingual frenum, an edge-to-edge bite, and a distally tipped left canine. The treatment objective was the closure of the diastema, achieving a normal overjet, normal overbite, and a class I molar relation and maintenance of class I canine relation and correcting the tip of the lower left canine, achieving a balanced smile and thus leading to an overall improvement of aesthetics. Figure 1 shows the diastema. Figure 2 shows the edge-to-edge bite.

**Figure 3:**
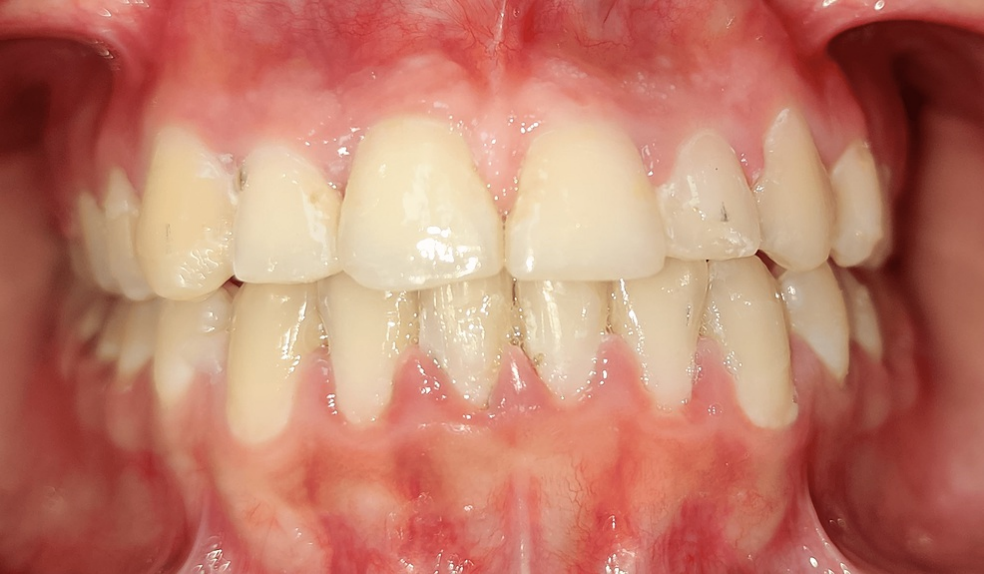
Closed midline diastema and the frenal attachment after frenectomy.

**Figure 4:**
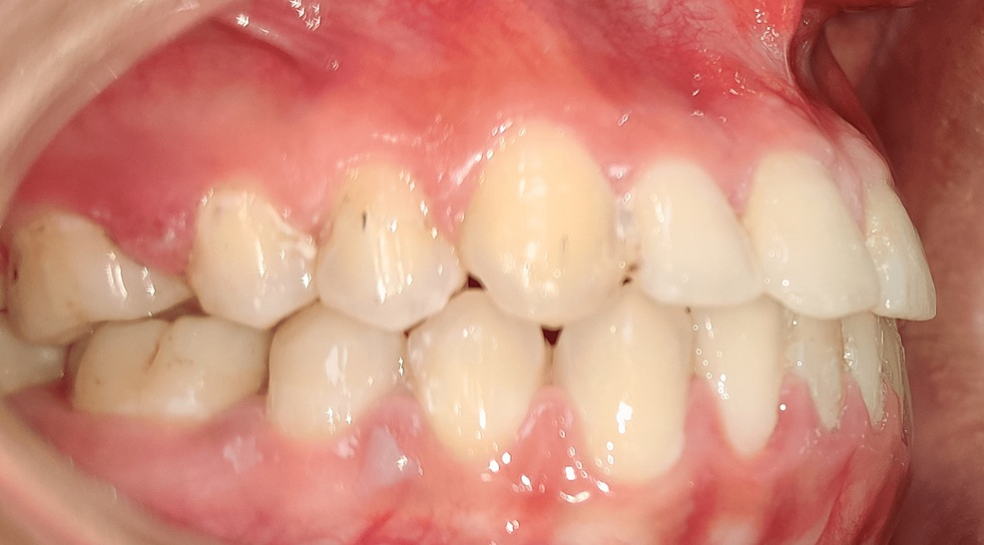
The improved overjet and overbite

The treatment plan was the closure of the space with continuous arch mechanics in the maxillary and mandibular arch and frenectomy of the labial frenum. The maxillary and mandibular arches were bonded using 0.018" pre-adjusted edgewise brackets [8]. The initial levelling and alignment were done with 0.014" NiTi, followed by 0.016" NiTi. Further 0.016" x 0.022" NiTi wire was placed. The levelling and alignment were completed after the placement of 0.017" x 0.025" stainless steel wire. Following this, the frenectomy was performed after taking the patient's informed consent. The frenectomy was performed under local anaesthesia using a number 15 BP blade [9]. An incision was made until the underlying bone was exposed. The area was sutured, and a periodontal pack was placed for seven days. The patient was recalled after a week for followup. Orthodontic treatment was commenced after one month of the frenectomy. The en-mass retraction was done using continuous arch mechanics. During this period of space closure, the patient was advised to use pink class III elastics. The overbite was corrected using a step-down bend in the 0.017" x 0.025" S.S. wire. After the space closure, finishing was done using settling elastics on 0.014" S.S. wire. At the end of the treatment, a class I molar and canine relation was achieved on both sides. The diastema was closed. Ideal overjet and overbite and overall improved aesthetics were achieved. Post-treatment retention was done through lingual bonded retainers [10]. The lingual bonded retainer was made of coaxial wire and was bonded to from canine to canine in both maxillary and mandibular arch. The total duration of the treatment was 10 months. Figure 3 shows the closed diastema. Figure 4 shows the ideal overjet and overbite. 

## Discussion

Midline diastema is considered a self-correcting anomaly at the age of 11 years [11-13]. It is present till the eruption of the permanent maxillary canine occurs. Seventeen percent of the diastema fails to close even after the eruption of permanent canines [3]. Spacing and diastema can occur because of microdontia, supernumerary teeth or, in the case of the present patient, abnormal frenal attachment. Although it is possible to treat spacing and diastema using composite restoration, the long-term success of such restoration is doubtful. Restorations undergo discolouration, and there are always chances of fractured restoration. Restorations can also lead to an unnecessary increase in the width of the teeth and must be done only in the case of microdontia.

Diastemas are most known for their high relapse tendency. Thus, a permanent lingual bonded retainer is the preferred retention method after its closure. The patient accepts it well because of its aesthetics. Methods to avoid relapse include correction of the aetiology. A frenectomy must be done in cases with abnormal frenal attachment [14]. Although performing a frenectomy before the commencement of orthodontic movement provides better surgical access, it is believed that the scar tissue formed due to the surgery may hamper the space closure [15]. Even after taking all the necessary care to avoid relapse, there are still chances of occurrence. Patients must be called for regular follow-up at least six months after the orthodontic treatment is complete.

## Conclusions

The present case presented in the case report shows the treatment of diastema with abnormal frenal attachment using continuous arch mechanics. Although it is challenging to retain the correction of diastema, using permanent retainers and establishing an excellent functional occlusion can reduce the chances of relapse. It is always important to treat the etiological factor which causes the malocclusion for a more stable result. In this case, the frenum, which was the leading cause of the diastema, was removed surgically, and a permanent retainer was given to reduce the chances of relapse.
